# Aberrant Inter-hemispheric Connectivity in Patients With Recurrent Major Depressive Disorder: A Multimodal MRI Study

**DOI:** 10.3389/fneur.2022.852330

**Published:** 2022-04-08

**Authors:** Guo Zheng, Zhang Yingli, Chen Shengli, Zhou Zhifeng, Peng Bo, Hou Gangqiang, Qiu Yingwei

**Affiliations:** ^1^Department of Hematology and Oncology, International Cancer Center, Shenzhen Key Laboratory, Hematology Institution of Shenzhen University, Shenzhen University General Hospital, Shenzhen University Health Science Center, Shenzhen University, Shenzhen, China; ^2^Department of Depressive Disorder, Shenzhen Kangning Hospital, Shenzhen Mental Health Center, Shenzhen, China; ^3^Department of Radiology, Huazhong University of Science and Technology Union Shenzhen Hospital, Shenzhen, China; ^4^Department of Radiology, Shenzhen Kangning Hospital, Shenzhen Mental Health Center, Shenzhen, China

**Keywords:** depression, multimodal MRI, interhemispheric connectivity MDD, major depressive disorder, functional connectivity, voxel-mirrored homotopic connectivity, corpus callosum

## Abstract

**Objective:**

Inter-hemispheric network dysconnectivity has been well-documented in patients with recurrent major depressive disorder (MDD). However, it has remained unclear how structural networks between bilateral hemispheres relate to inter-hemispheric functional dysconnectivity and depression severity in MDD. Our study attempted to investigate the alterations in corpus callosum macrostructural and microstructural as well as inter-hemispheric homotopic functional connectivity (FC) in patients with recurrent MDD and to determine how these alterations are related with depressive severity.

**Materials and Methods:**

Resting-state functional MRI (fMRI), T1WI anatomical images and diffusion tensor MRI of the whole brain were performed in 140 MDD patients and 44 normal controls matched for age, sex, years of education. We analyzed the macrostructural and microstructural integrity as well as voxel-mirrored homotopic functional connectivity (VMHC) of corpus callosum (CC) and its five subregion. Two-sample *t*-test was used to investigate the differences between the two groups. Significant subregional metrics were correlated with depression severity by spearman's correlation analysis, respectively.

**Results:**

Compared with control subjects, MDD patients had significantly attenuated inter-hemispheric homotopic FC in the bilateral medial prefrontal cortex, and impaired anterior CC microstructural integrity (each comparison had a corrected *P* < 0.05), whereas CC macrostructural measurements remained stable. In addition, disruption of anterior CC microstructural integrity correlated with a reduction in FC in the bilateral medial prefrontal cortex, which correlated with depression severity in MDD patients. Furthermore, disruption of anterior CC integrity exerted an indirect influence on depression severity in MDD patients through an impairment of inter-hemispheric homotopic FC.

**Conclusion:**

These findings may help to advance our understanding of the neurobiological basis of depression by identifying region-specific interhemispheric dysconnectivity.

## Introduction

Depression is a common mental disorder and a leading cause of disability, affecting more than 264 million people worldwide (1). Although it is highly prevalent, the precise neurobiological mechanisms underlying major depressive disorder (MDD) are still not fully elucidated (2). Convergent evidence from neuroimaging studies has suggested that patients with depression exhibit structural and functional abnormalities in cerebral regions or networks that participate in affective regulation (2–5).

Bihemispheric communication plays a vital role in integrating neural circuits and underlies coherent cognition, emotional regulation, and behavior (6, 7). Interhemispheric functional homotopy, reflecting bihemispheric communication, has been verified as one of the most salient and stable features of the brain's intrinsic functional infrastructure (8). Impairment of inter-hemispheric functional homotopy has been wildly documented in MDD patients via functional magnetic resonance imaging (fMRI) (7, 9–13). However, the results of such fMRI studies have been somewhat inconsistent. Interhemispheric functional dysconnectivity has been observed in the bilateral posterior default mode network (DMN) in first-episode medication-naïve MDD patients (9, 12), in the bilateral sub-genu anterior cingulate cortex in resilient MDD patients (14), and in the bilateral cuneus, putamen, superior temporal gyrus, insula, and precuneus in patients with current and recurrent MDD (11). Even in first-episode patients without treatment, some studies have implicated that interhemispheric dysconnectivity is mainly involved in the anterior subnetwork of the DMN (10, 15), while other studies have reported the posterior DMN (9, 12), insular cortex, and putamen (13) as being more vulnerable to MDD. These inconsistent and conflicting findings must be interpreted with caution, as most previous studies have only recruited relatively small sample sizes of MDD patients (9, 10, 13). Furthermore, the underlying structural mechanisms of altered inter-hemispheric functional connectivity (FC) in patients with MDD remain largely unknown (11).

The corpus callosum (CC) is the largest interhemispheric commissure and plays a cardinal role as the primary cortical projection system connecting the two brain hemispheres (16). CC degeneration, in terms of either volumetric reduction or diffusivity disruption, has been documented in MDD (12, 17, 18). However, functionally, the CC consists of different components that bridge different brain regions (16), The genu of the CC connects the orbitofrontal and frontal cortices, whereas the body and the splenium connects the temporal, parietal, and occipital regions (19). Therefore, it is important for further studies to determine which CC subfields are the most susceptible to depression, as well as the relationships among regional-specific CC impairment, inter-hemispheric homotopic functional dysconnectivity, and depression severity in MDD patients.

To address these questions, we combined high-resolution structural MRI, diffusion tensor MRI, resting-state functional MRI, and the Hamilton Depression Rating Scale (HDRS) to investigate disruption of inter-hemispheric homotopic FC, CC degeneration (and its subregions), and depression severity in a relatively large sample of recurrent MDD patients. We hypothesized that structural degeneration in the anterior CC (compared to that of the posterior CC) may be associated with bilateral prefrontal cortex dysconnectivity, which may underly depression severity in MDD.

## Methods and Materials

### Patients

We recruited 140 patients with recurrent MDD, all the patients were currently experiencing an episode of major depression, but had not receive standardized drug treatment under guidance of doctor. Patients were diagnosed according to the Structured Clinical Interview for the Diagnostic and Statistical Manual of Mental Disorders (DSM-IV) (SCID). Depression severity was measured using the 17-item HDRS. Inclusion criteria for individuals with MDD included the following: aged 16–70 years, right-hand dominant, and met the DSM-IV diagnostic criteria for MDD with a current depressive episode. Healthy controls were included if they met the following criteria: aged 16–70 years, right-hand dominant, and no personal or family history of major psychiatric illness.

Exclusion criteria were as follows: a history of another DSM-IV Axis-I disorder (e.g., schizophrenia, schizoaffective disorder); any other physical disease affecting brain function (e.g., brain trauma, epilepsy, stroke); a history of substance dependence or abuse; or other contraindications for undergoing an MRI (e.g., serious medical illness, breastfeeding, pregnancy).

Written informed consent was acquired from each participant before study enrollment. The study protocols were approved by the Institutional Review Board of the Medical Ethics Committee of Shenzhen Kangning Hospital.

### Clinical and Cognitive Outcome Measures

Symptom severity for each patient was assessed with the 17-item HDRS (20, 21). Neuropsychological assessments were conducted by research personnel, who were trained and supervised by a registered clinical neuropsychologist (Yingli Zhang).

### MRI Acquisition

All MRI data were acquired on a 3.0-Tesla scanner (Discovery MR750 System; General Electric, Milwaukee, WI, USA) with an 8-channel head coil. Each participant was asked to lay in a supine position with his or her head snugly fixed by a belt and foam pads, and earplugs were used to reduce noise. Before MRI scanning was initiated, participants were required to keep their eyes closed, to not fall asleep, and remain motionless during the scanning period. First, a T1-weighted image (repetition time [TR] = 1,750 ms, echo time [TE] = 24 ms) and T2-FLAIR images (TR = 8,400 ms, TE = 120 ms, and inversion time [TI] = 2,100 ms) were captured to exclude subjects with abnormalities. Then, a resting-state fMRI scan with an echo-planar imaging (EPI) sequence, a high-resolution structural MRI scan with a fast-field echo (FFE) three-dimensional T1-weighted (3D-T1WI) sequence, and a diffusion tensor MRI scan with a single-shot spin-echo-planar sequence were sequentially conducted. Detailed imaging parameters were as follows: (1) resting-state fMRI: TR/TE = 2,000/30 ms, flip angle = 90°, thickness/gap = 3.5/0 mm, acquisition matrix = 64 × 64, field of view (FOV) = 224 × 224 mm^2^, 33 axial slices, and 240 time points (8 min); (2) 3D-T1WI: TR/TE = 6.65/2.93 ms, flip angle = 12°, acquisition matrix = 256 × 256, FOV = 256 × 256 mm^2^, and 192 sagittal slices with no inter-slice gap; (3) diffusion tensor MRI: 64 diffusion-weighted images (b = 1,000 s/mm^2^) and 10 non-diffusion-weighted images (b = 0 s/mm^2^) using a spin-echo echo-planar imaging sequence with the following parameters: number of excitations = 1, repetition time (TR) = 8,724 ms, echo time (TE) = 81.4 ms, acquisition matrix = 112 × 112, and voxel size = 2 mm × 2 mm × 2 mm.

### Preprocessing of Structural Images and CC Segmentation

We preprocessed structural MRI data according to the FreeSurfer pipeline (version 6.0, http://surfer.nmr.mgh.harvard.edu), according to previous studies (22, 23). Manual editing was performed where appropriate, and scans with unresolvable reconstruction errors were excluded from further analysis. The CC was automatically identified and segmented into five segments according to our previously published protocol (23–25), with each section representing a fifth of the total area/volume that broadly corresponds to the following functional subdivisions (26): anterior (CC1), mid-anterior (CC2), central (CC3), mid-posterior (CC4), and posterior portions (CC5; Supplementary Figure 1). The total CC volume was calculated as the sum of the five segments.

### Preprocessing of Diffusion Tensor MRI Data and Derivation of CC Subregional Microstructure

FreeSurfer's dt_recon program was used to preprocess the diffusion tensor MRI data. First, eddy-current compensation and motion-correction procedures were conducted on diffusion volumes using FLIRT (27). Non-brain tissues were then removed from the diffusion-weighted imaging using the Brain Extraction Tool (BET) from FSL (28). Next, general linear model (GLM) fitting and tensor construction were conducted to generate fractional anisotropy (FA) maps (29). Thereafter, low b-diffusion images were registered to the same-subject anatomical coordinates using a boundary-based registration procedure (30). Finally, the above-generated FA maps were mapped to talairach space (31). Then the average FA values of the CC subfields were extracted through mri_segstats. The mean head-motion parameters for translation and rotation were also computed for each subject and were included as a covariate in statistical analyses. Details can be found at http://surfer.nmr.mgh.harvard.edu/fswiki/dt_recon#dtrecon.

### Preprocessing of Resting-State FMRI Data and Derivation of Interhemispheric FC

We preprocessed the resting-state fMRI data using Data Processing & Analysis of Brain Imaging (DPABI 1.2) (32), which is based on statistical parametric mapping (SPM8, http://www.fil.ion.ucl.ac.uk/spm), following our previous approaches (24, 25, 33). Details regarding our preprocessing of resting-state fMRI data can be found in the Supplementary Materials. After quality control, all participants met the criterion of minimum absolute translation and rotation not exceeding 1.5 mm or 1.5°. Moreover, the mean framewise displacement (FD) was computed by averaging the FD from every time point for each subject, and it was included as a covariate in statistical analyses (34).

We computed subject-level interhemispheric FC by using voxel-mirrored homotopic connectivity (VMHC) method, which calculated the functional connectivity between each voxel in one hemisphere and its mirrored counterpart in the other following previous research (35). Shortly speaking, for every subject, Pearson's correlation was used to obtain the homotopic FC between each voxel's residual time series and that of its mirrored inter-hemispheric counterpart. Thereafter, correlation values were Fisher's z-transformed to produce subject-specific VMHC z-score maps for further statistical analyses.

### Statistical Analysis

Categorical variables were inspected using chi-squared tests, and continuous variables were compared using two-sample *t*-tests or non-parametric tests.

CC subfield volumes and FA were compared via univariate analysis in SPSS version 26.0 (IBM Inc., Armonk, NY). Analyses were two-tailed and evaluated for significance at the 0.05 alpha level (controlling for age, gender, education, TIV [for volume], and head motion [for FA]). Multiple-comparison corrections were performed using Bonferroni corrections.

Interhemispheric FC differences between groups were assessed by independent-sample *t*-tests in SPM v12 (Wellcome Trust Centre for Neuroimaging, UCL, London, UK), and nuisance covariates included age, gender, education, and head motion. The results were reported at a height threshold of *P* < 0.01 and a cluster threshold of *P* < 0.05, with a Gaussian random field (GRF) correction.

## Results

### Demographic Information

There were no group differences in terms of age, sex distribution, education, or head motion (Table 1). For MDD patients, the median disease duration for MDD was 3.35 years, and the median HDRS score was 22.

**Table 1:** Demographic and clinical characteristics of patients with recurrent major depressive disorder (MDD) and healthy controls.

**Demographic characteristics**	**MDD patients (*n* = 140)**	**Healthy controls (*n* = 44)**	**χ2/z**	***P***
Gender (male/female)	41/99	17/27	1.356	0.244_a_
Age (y)	33 (24,48)	38.5 (27.25,56.25)	−1.712	0.087_b_
Education (y)	15 (12,17)	15 (12,17)	−1.627	0.104_b_
Duration of depressive episode (y)	3.35 (1.05,10.00)	–		
Depression severity (HDRS)	22 (18,27)	–		
**DTI**				
Head-motion Translation (mm)	0.986 (0.878, 1.143)	0.947 (0.874, 1.158)	−0.133	0.894_b_
Rotation (°)	0.007 (0.006, 0.009)	0.007 (0.006, 0.010)	−0.493	0.422_b_
^*^Rs-fMRI head motion	0.117 (0.081, 0.171)	0.126 (0.098, 0.173)	−0.97	0.332_b_

*Unless otherwise indicated, data are expressed as P_50_ (P_25_, P_75_)*.

a *P value obtained by chi-squared test*.

b *P value obtained by Mann-Whitney test*.

* *Rs-fMRI head motion was indexed by mean framewise displacement (FD) derived with Jenkinson's relative root mean square (RMS) algorithm*.

### Disruption of Anterior CC Microstructural but Not Macrostructural Integrity in MDD Patients

**Figure 1:**
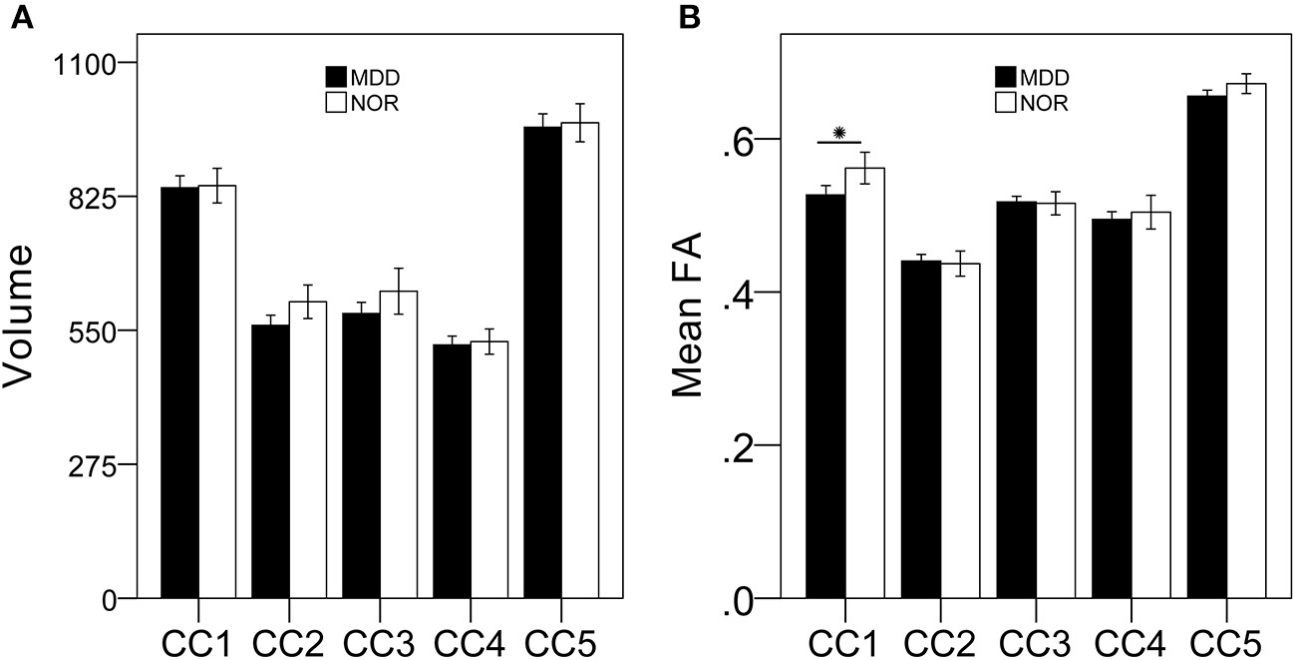
MDD patients exhibit damaged anterior CC microstructural but not macrostructural integrity compared with that of healthy controls. There were no significant differences in CC subregional volumes between MDD patients and healthy controls **(A)**; MDD patients had lower FA of the anterior CC subregion compared to that of healthy controls **(B)**. *p < 0.05, bonferroni corrected.

MDD patients had lower FA of the anterior CC, but a relatively stable volume, when compared with these parameters in healthy controls, suggesting that MDD patients exhibit microstructural integrity disruption in the anterior CC (Figure 1).

### Disruption of Inter-hemispheric Homotopic FC of the Bilateral Medial Prefrontal Cortex in MDD Patients

**Figure 2:**
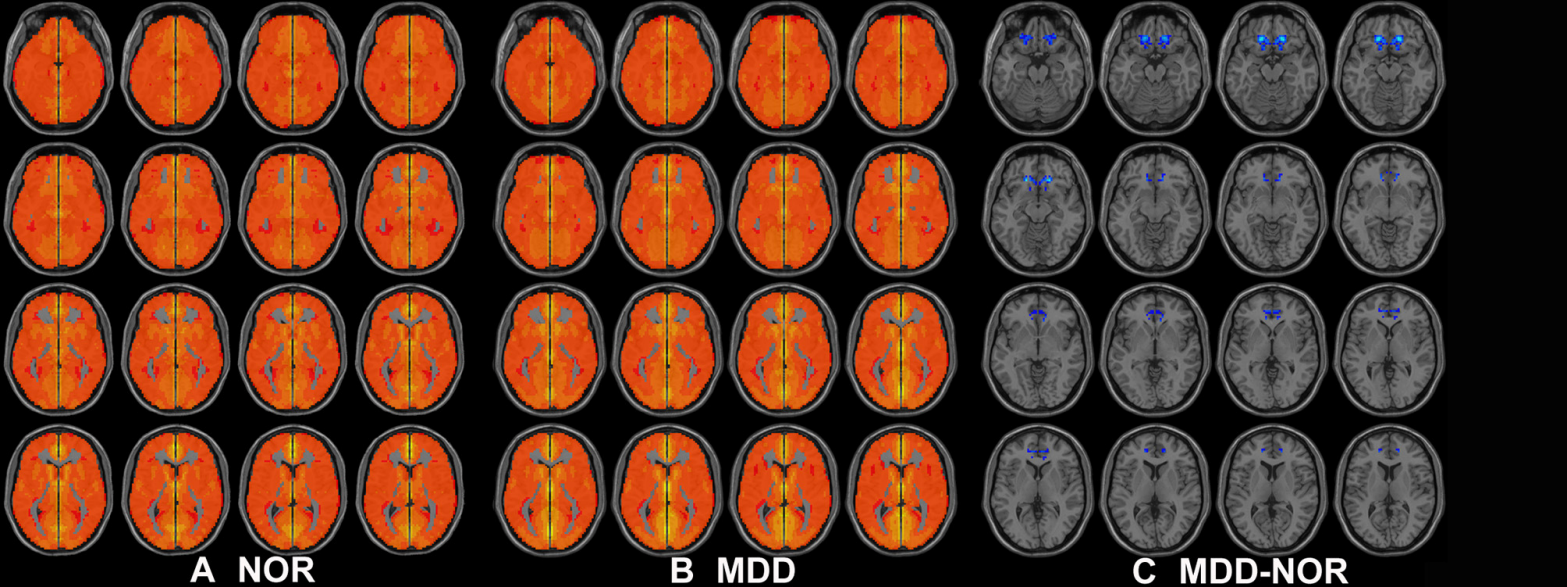
Axial MRI scans show interhemispheric FC within and between groups. Regions show significant interhemispheric FC in **(A)** healthy controls and **(B)** MDD patients (*P* < 0.05, AlphaSim corrected). **(C)** Homotopic regions show decreased (cool color) FC in the MDD group (*P* < 0.05, AlphaSim corrected).

Compared to that in healthy controls, MDD patients showed a significant decrease in inter-hemispheric FC in the bilateral medial prefrontal cortex (Figure 2, Table 2).

**Table 2:** Group differences in inter-hemispheric FC between healthy controls and MDD patients.

**Regions**	**BA**	**MNI Coordinates**	**Peak t-score**	**Cluster size (mm^**3**^)**
		X	Y	Z		
Medial FG/ACC/OFG/IFG	10, 11, 25, 32, 47	18	30	−15	−4.3899	4,266

*Statistical significance was set at a height threshold of P < 0.01 and cluster threshold of P < 0.05 with GRF correction*.

*BA, Broadman area; FG, frontal gyrus; ACC, anterior cingulate cortex; OFC, orbitofrontal cortex; IFG, inferior frontal gyrus*.

### Bilateral Medial Prefrontal Dysconnectivity Correlates With Disruption of Anterior CC Microstructural Integrity and Depression Severity in MDD Patients

**Figure 3:**
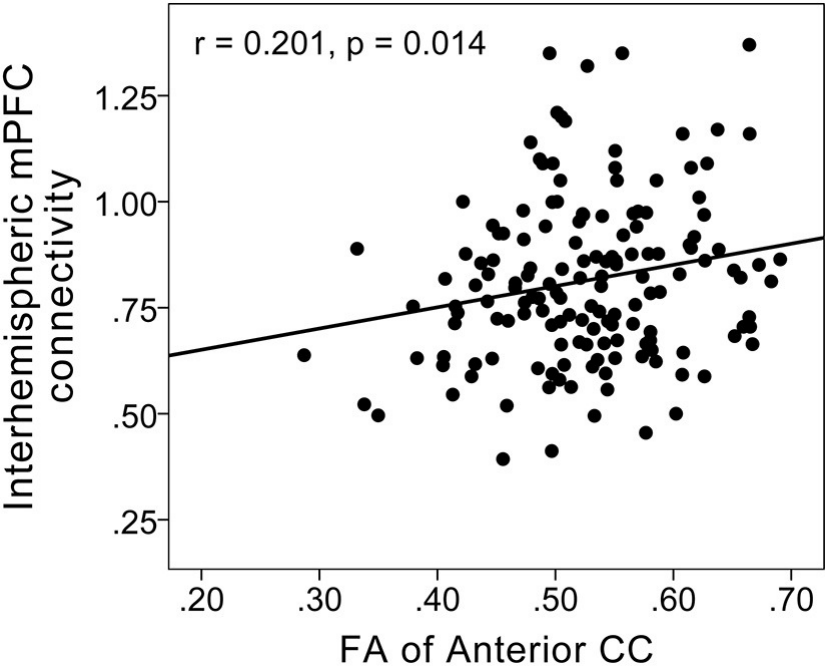
Scatterplots showing a positive correlation between FC in the bilateral medial prefrontal cortex and fractional anisotropy of the anterior CC in MDD patients. Interhemispheric homotopic FC of the bilateral medial prefrontal cortex correlated with anterior CC microstructural integrity (r = 0.201; *P* = 0.014).

**Figure 4:**
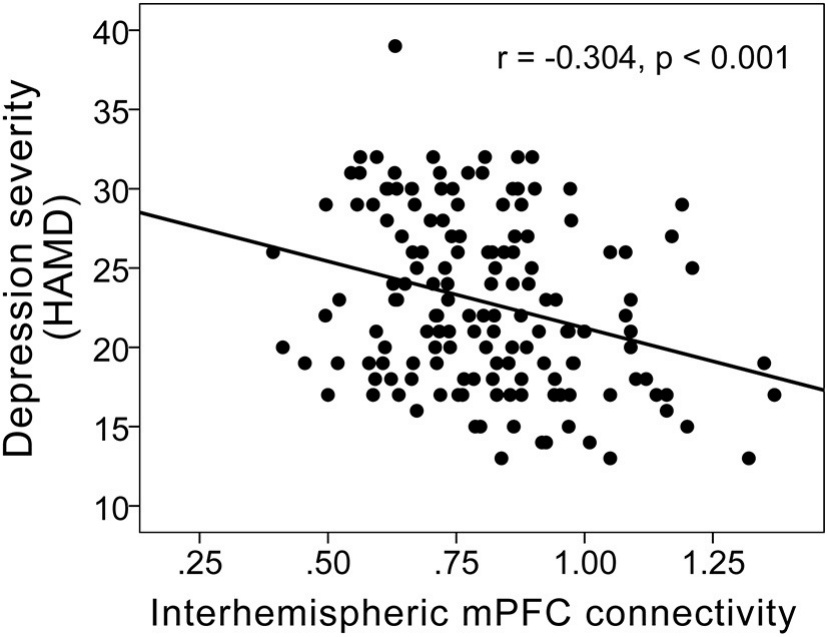
Scatterplots showing a negative correlation between FC of the bilateral medial prefrontal cortex and depression severity (r = −0.304; *P* < 0.001) in MDD patients.

Interhemispheric homotopic FC of the bilateral medial prefrontal cortex correlated with anterior CC microstructural integrity (r = 0.201; *P* = 0.014) (Figure 3) and depression severity (r = −0.304; *P* < 0.001) in MDD patients (Figure 4).

### Disruption of the Anterior CC Has an Indirect Influence on Depression Severity in MDD Patients

**Figure 5:**
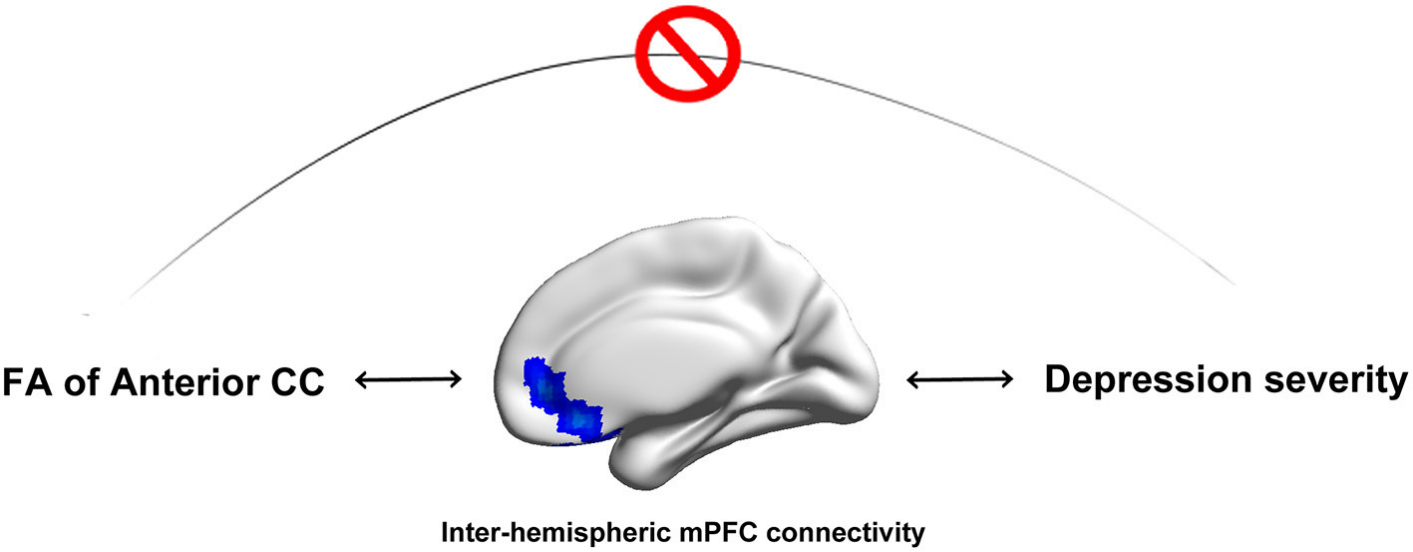
Anterior CC microstructural integrity has an indirect influence on depression severity in MDD patients. Disruption of anterior CC microstructural integrity has an indirect impact (through exerting effects on FC of the bilateral medial prefrontal cortex) on depression severity in MDD patients.

Anterior CC disruption had an indirect impact (through exerting effects on FC of the bilateral medial prefrontal cortex) on depression severity in MDD (Figure 5).

## Discussion

To the best of our knowledge, our present study represents the first report of reduced interhemispheric homotopic FC and structural connectivity (evaluated by CC and its subregional macrostructure and microstructure) in MDD patients, as elucidated via multimodal MRI. A primary finding of the present study was significantly decreased VMHC in the bilateral medial prefrontal cortex, which correlated with disruption of anterior CC (genu) microstructural but not macrostructural integrity in MDD patients. Further analysis revealed that severe depressive symptoms (higher HDRS scores) correlated with decreased interhemispheric FC of the bilateral medial prefrontal cortex in MDD patients. Moreover, aberrant anterior CC integrity exerted an indirect effect on depression severity in MDD patients, which was associated with reduced FC in the bilateral medial prefrontal cortex. Taken together, these findings may help to advance our understanding of the neurobiological basis of depression by identifying region-specific inter-hemispheric dysconnectivity in MDD patients.

### Selectively Anterior CC Microstructural but Not Macrostructural Damage in MDD Patients

It is noteworthy that MDD patients had selective impairment of anterior CC microstructural integrity, but not of any other CC subfields, relative to matched healthy controls. This finding is not surprising given that the anterior CC bridges bilateral prefrontal and orbitofrontal regions (36), which are central to current theories of mood regulation in humans (37, 38). Disruption of anterior CC microstructural integrity, implying decreased structural connectivity between prefrontal and orbitofrontal regions, may constitute a common pathway underlying mood dysregulation in MDD (38). Selective anterior CC damage is a well-replicated finding in MDD patients from previous studies (17, 18, 38, 39). Using a region-of-interest method, Dillon et al. found that MDD patients had reduced FA in the genu of the CC, in addition to lower FA in the anterior limb of the internal capsule, the cingulum bundle near the ACC, and the uncinate fasciculus. Moreover, they demonstrated an inverse relationship between depressive severity and FA in the genu of the CC (r = −0.44, *P* < 0.01) (39). Xu et al. also found that FA values were significantly lower in the anterior genu of the CC in the medication-free MDD group than in the control group (*P* = 0.009, corrected), while no significant differences were found in any other CC subregions (17). In a meta-analysis of medication-free patients with MDD, Jiang et al. found that only medication-naive patients with MDD displayed FA reductions in the genu of the CC and in right anterior thalamic projections (18). Similarly, another meta-analysis also showed that patients with major depression and bipolar disorder were characterized by abnormalities in white matter tracts of the genu of the CC that connect the two hemispheres of the prefrontal cortex implicated in mood regulation (36). Taken together, these findings suggest that disruption of anterior CC microstructural integrity, implying structural dysconnectivity between prefrontal and orbitofrontal regions, may be an underlying neurobiological mechanism of mood dysregulation in MDD.

Unexpectedly, in our present study, volumetric measurements of the CC and its subfields did not differ between MDD patients and matched healthy controls. In contrast to the consistent results from studies of CC microstructural integrity, studies of CC volumetric measurements have yielded inconsistent results in MDD patients (12). Ran et al. revealed that patients with MDD had smaller volumes in the genu of the CC than in controls, as determined by FreeSurfer segmentation (12). In addition, Emsell et al. did not find any differences in CC subregional thickness or surface area (by dividing the mid-sagittal CC into seven subregions); thus, they demonstrated that callosal structure was largely preserved in late-life depression (40). Ozalay et al. also demonstrated that there were no differences in the areas or lengths of the total CC or any CC subfields (by dividing the CC into five parts) between MDD patients and healthy controls. Our present findings are in agreement with most previous studies (41). These findings suggest that CC microstructural alterations are more sensitive to changes in tissues than are volumetric measurements, and may predate macrostructural changes in MDD. Therefore, a reduction in anterior CC microstructural integrity (but not macrostructural integrity) may serve as an *in vivo* biomarker of MDD.

### Interhemispheric Functional Dysconnectivity in the Medial Prefrontal Cortices of MDD Patients

As expected, we found that MDD patients exhibited reductions in inter-hemispheric homotopic FC in the medial prefrontal cortex, which extends to the anterior cingulate cortex and orbitofrontal cortex, the cardinal regions responsible for emotional regulation (37). This finding is consistent with previous pathology-based evidence of prefrontal abnormalities in MDD patients (42, 43) and multimodal imaging models (2, 11, 44, 45). The medial prefrontal cortex is a key part of the cortico-striato-thalamo-cortical loop, with multiple connections to the limbic system (including the amygdala and hippocampus), basal ganglia, and thalamus. Given this extensive network, the medial prefrontal cortex and ACC have been postulated to be functional in the monitoring and regulation of emotional states; thus, dysconnectivity of the bilateral medial prefrontal cortex may be implicated in mood dysregulation in MDD patients.

Furthermore, we found that FC in the bilateral medial prefrontal cortex was positively correlated with FA of the anterior CC, and was inversely correlated with depression severity (HDRS scores), indicating that anterior CC microstructural disruption may represent the structural basis of dysconnectivity in the bilateral prefrontal cortex, and that reduced FC in the bilateral prefrontal cortex may represent depression severity in MDD patients. Furthermore, we found that anterior CC microstructural deficits exerted an indirect effect on depression severity in MDD patients, which was associated with reduced FC in the bilateral medial prefrontal cortex, suggesting that white matter microstructural disruptions the anterior CC itself may not directly exert effects on depression, but that impaired anterior CC white matter integrity may lead to an imbalance in frontal cortical networks, which in turn may confer depression.

Despite the strengths of our present study—including its relatively large sample size and integrated use of multimodal MRI methods—there were also some limitations. First, although current DTI analysis revealed anterior CC subfield's microstructural integrity was impaired in MDD patients, the present results should be interpreted with caution, given the contamination of neuroinflammation as well as crossing fiber should be considered. Thus, free water corrected-DTI analysis as well as advanced diffusion imaging, such as diffusion spectrum imaging (46) and high-angular resolution diffusion imaging (47), should be conducted to validate these preliminary findings in future. Second, as a cross-sectional study, we can only observe the interaction effects among impairment of anterior CC integrity, dysfunctional of inter-hemispheric homotopic FC of the bilateral medial prefrontal cortex and depression severity in MDD patients. However, we cannot absolutely assert the causation among them. Longitudinal are needed in future. Third, the included MDD patients were clinically heterogeneous in terms of illness duration, age of onset, number of episodes, and prior medication history. These factors may have weakened our sensitivity in revealing MDD-related alterations in the CC and its subregional volumes. Forth, although we integrated multiple imaging modes (structural, diffusion, and resting-state BOLD) in the present study, we only focused on inter-hemispheric structural and functional aberrations in MDD patients. In future, other quantitative indices (e.g., whole-brain network-related connectivity analysis; voxel/vertex-based morphological analysis) should be investigated in future studies to further elucidate the pathogenesis of MDD. Fifth, VMHC methods may have inherent limitations due to the bilateral hemispheres not being entirely symmetric. However, spatial smoothing was applied to improve the spatial correspondence between homotopic areas and to reduce the impact of the asymmetric nature of the brain, which can also minimize the potential confounder of inter-subject anatomical variability. Last, the CC was equally sub-divided with the FreeSurfer method, which might not accurately reflect the true fiber compositions in each subregion.

In conclusion, we investigated interhemispheric homotopic FC and structural connectivity in patients with MDD via multimodal MRI. An impairment in interhemispheric FC correlated with clinical depression severity in MDD patients. Furthermore, disruption of anterior CC integrity exerted an indirect effect on depression severity in MDD patients. Our findings provide new insights into the neural mechanisms of MDD and reinforce an integrative view of the functional and structural organization of the interhemispheric brain in MDD patients. These findings may help to advance our understanding of the neurobiological basis of depression by identifying region-specific interhemispheric dysconnectivity in MDD patients.

## Data Availability Statement

The original contributions presented in the study are included in the article/Supplementary Material, further inquiries can be directed to the corresponding author/s.

## Ethics Statement

The studies involving human participants were reviewed and approved by the Institutional Review Board of the Medical Ethics Committee of Shenzhen Kangning Hospital. Written informed consent to participate in this study was provided by the participants' legal guardian/next of kin. Written informed consent was obtained from the individual(s) for the publication of any potentially identifiable images or data included in this article.

## Author Contributions

QY and HG conception and design. GZ, ZY, CS, ZZ, and PB collection and assembly of the data. QY development of the methodology. All authors: data analysis and interpretation, manuscript writing, and final approval of the manuscript.

## Funding

This work was supported by the grants from the Natural Scientific Foundation of China (grant numbers: 81560283 and 81201084), the Guangdong Basic and Applied Basic Research Foundation (2020A1515011332), the Shenzhen Sanming Project (No. SZSM201512038), and the Shenzhen Fund for Guangdong Provincial High-level Clinical Key Specialties (No. SZGSP013), Shenzhen Key Medical Discipline Construction Fund (No. SZXK041), Shenzhen Science and Technology Foundation (JCYJ20190808163601776, JCYJ20200109113810154), Shenzhen Key Laboratory Foundation (ZDSYS20200811143757022), and Sanming Project of Medicine in Shenzhen (SZSM202111004). The funding source of the study had no role in the study design, data collection, data analysis, data interpretation, writing of this report, or decision to submit for publication. The corresponding author had full access to all data in the study and had final responsibility for the decision to submit for publication.

## Conflict of Interest

The authors declare that the research was conducted in the absence of any commercial or financial relationships that could be construed as a potential conflict of interest.

## Publisher's Note

All claims expressed in this article are solely those of the authors and do not necessarily represent those of their affiliated organizations, or those of the publisher, the editors and the reviewers. Any product that may be evaluated in this article, or claim that may be made by its manufacturer, is not guaranteed or endorsed by the publisher.
