# Supplementary material for: Aberrant Inter-hemispheric Connectivity in Patients With Recurrent Major Depressive Disorder: A Multimodal MRI Study
Source: Front Neurol. 2022 Apr 8;13:852330. doi: 10.3389/fneur.2022.852330 (PMC9028762; doi:10.3389/fneur.2022.852330)
Supplement: Supplementary file 1 [file Data_Sheet_1.docx]

**Aberrant Inter-hemispheric Connectivity in Patients with Recurrent Major Depressive Disorder: a Multimodal MRI Study**

***Preprocessing of resting-state fMRI data***

Details for preprocessing of resting-state fMRI data were as follows: (1) removal of the first 10 time-points to allow magnetization to reach steady state; (2) slice-timing correction and head motion correction; (3) Normalized the functional images to the MNI-152 template using non-linear transformation; (4) Smoothed the functional imaging using a 6 mm full width high maximum (FWHM) Gaussian kernel; (5) removal the nuisance variable (e.g., cerebral spinal fluid, motion parameters); and finally (6) the signal was linearly detrended and bandpass filtered at 0.01–0.08 Hz to reduce the effect of low-frequency drifts and high-frequency uninteresting signals.

**Supplementary Figure 1. Schematic of corpus callosum segmentation into five equal segments.**

**
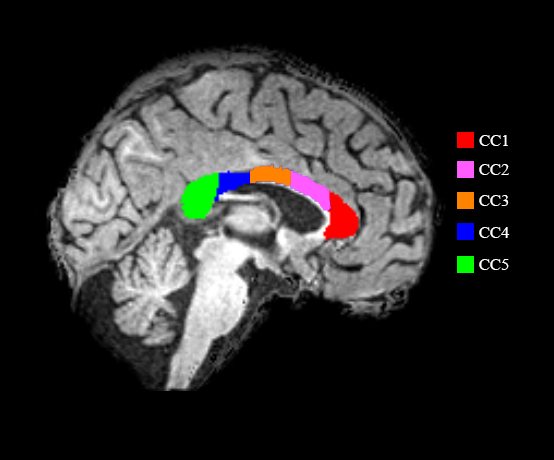
**

CC, corpus callosum; CC1 (anterior), rostrum; CC2 (mid-anterior), genu; CC3 (middle), truncus/body; CC4 (mid-posterior), anterior splenium; CC5(posterior portions), roughly equivalent of the posterior splenium
